# Subjective Cognitive Fatigue in Multiple Sclerosis Depends on Task Length

**DOI:** 10.3389/fneur.2014.00214

**Published:** 2014-10-27

**Authors:** Joshua Sandry, Helen M. Genova, Ekaterina Dobryakova, John DeLuca, Glenn Wylie

**Affiliations:** ^1^Neuropsychology and Neuroscience Research, Kessler Foundation, West Orange, NJ, USA; ^2^Department of Physical Medicine and Rehabilitation, Rutgers – New Jersey Medical School, Newark, NJ, USA; ^3^Department of Neurology and Neurosciences, Rutgers – New Jersey Medical School, Newark, NJ, USA; ^4^War Related Illness and Injury Study Center, Department of Veterans Affairs, East Orange, NJ, USA

**Keywords:** cognitive fatigue, fatigability, time, processing speed, working memory

## Abstract

**Objective:** The objective of this paper is to investigate the interrelationship between subjective and objective cognitive fatigue, information processing domain [processing speed (PS) vs. working memory (WM)], cognitive load (high vs. low), and time on task in Multiple Sclerosis (MS).

**Methods:** Thirty-two MS participants and 24 healthy controls completed experimental tasks in both the PS and WM domains with different levels of cognitive load. Subjective cognitive fatigue was measured using a visual analog scale at baseline and at multiple time points throughout the experiment.

**Results:** A mixed model ANOVA revealed that subjective cognitive fatigue was higher for the PS task, increased across time, and was higher in the MS group. These findings were qualified by an interaction demonstrating that the MS group showed a steeper increase in subjective cognitive fatigue over time than the healthy control group. Subjective and objective (i.e., performance) cognitive fatigue were not correlated.

**Conclusion:** In this study, subjective and objective cognitive fatigue appears to be independent and cognitive fatigue does not depend on cognitive load. Subjective cognitive fatigue increased with time on task and subjective cognitive fatigue increased more steeply for the MS group. These data suggest that cognitive fatigue in MS is a function of time, that is, the longer participants were engaged in a cognitive task, the more likely it was for them to report increases in cognitive fatigue.

## Introduction

Fatigue is perhaps the most common complaint associated with Multiple Sclerosis (MS) (1) with prevalence estimates ranging between 70 and 90% (2–4). Fatigue can be cognitive or motoric and originate at a central level (i.e., the central nervous system) or peripheral level (i.e., peripheral nerve and muscle) (5). Cognitive fatigue can be a result of both cognitive and physical exertion (6). Cognitive fatigue may manifest as subjective sensations or objective changes in performance, fatigue, and fatigability, respectively (7). Treating cognitive fatigue clinically remains difficult, particularly because a basic understanding of the variables that contribute to cognitive fatigue are not well defined. The present paper investigates the task parameters that lead to cognitive fatigue in MS. This knowledge may help to inform future research as well as clinical evaluations of cognitive fatigue in MS. Novel insights into how and why cognitive fatigue manifests may also ultimately lead to improved clinical treatment strategies for cognitive fatigue.

One strong predictor of cognitive fatigue is the amount of time spent on task (time on task); as time on task increases cognitive fatigue also increases (8–11). Interestingly in some instances increased time on task can improve performance (12, 13). Reports are mixed regarding the effect of time on cognitive fatigue in MS. Some researchers have shown that time on task may result in increases in subjective cognitive fatigue but not decreases in objective performance (14). Others have shown time negatively impacts both subjective and objective measures (15). Interestingly, most studies of cognitive fatigue in MS have failed to show a relationship between subjective and objective measures of cognitive fatigue (9, 14–18). Further, high and low levels of fatigue do not map onto changes in cognitive performance (19). Cognitive load is an additional variable to consider when investigating cognitive fatigue.

Tasks high in cognitive load (challenging tasks) often result in greater increases in subjective cognitive fatigue than tasks low in cognitive load (less challenging tasks) (20). High cognitive load can also result in a more rapid onset of subjective cognitive fatigue (21). In past work, Bailey et al. (16) tested the consequences of cognitive load and time on task for cognitive fatigue in a sample of advanced MS participants [Expanded Disability Status Scale (EDSS) of 7–8]. Researchers manipulated high and low cognitive load using the n-back working memory (WM) task, 0-back, and 1-back, respectively. Subjective cognitive fatigue increased across sessions for the high cognitive load condition in the MS and HC groups and this effect was more pronounced for the MS group, suggesting that patients with advanced stages of MS are more likely to experience cognitive fatigue on challenging tasks.

One unanswered question is whether MS patients are more susceptible to cognitive fatigue in one cognitive domain than in a different cognitive domain. For example, some evidence suggests that impaired processing speed (PS) is the major information processing deficit associated with MS (22, 23) while other evidence suggests that impaired WM is the major information processing deficit associated with MS (24). Based on this past work, it may be the case that tasks that engage different cognitive domains result in different patterns of cognitive fatigue. Cognitive fatigue may be domain specific and when one domain is impaired, e.g., PS, more neural resources must be recruited from other domains. Expending more resources could result in patients reporting higher levels of cognitive fatigue. At this point, it remains unclear how cognitive fatigue manifests in MS as a function of different cognitive domains and different degrees of cognitive load associated with those domains. What is also unclear is how time, arguably the strongest predictor of cognitive fatigue, interacts with these variables.

The purpose of the present study was to examine whether cognitive fatigue (both objective and subjective) is influenced by type of cognitive task (i.e., PS vs. WM) in MS. Based on the reviewed literature, three competing hypotheses that influence cognitive fatigue can be directly tested: (1) *the cognitive load hypothesis* (16), (2) *the cognitive domain hypothesis* (22–24), and (3) *the temporal fatigue hypothesis* (8–11). Particularly strong support for any hypothesis and its relationship with MS will come from an interaction between group and the related main effect. If the Cognitive Load hypothesis is correct, then reported fatigue will be higher as a function of task difficulty: higher reported fatigue in the high cognitive load conditions compared to lower reported fatigue in the low cognitive load conditions. If the Cognitive Domain hypothesis is correct, there will be higher reported fatigue in a particular information processing domain (PS or WM). If the Temporal Fatigue hypothesis is correct, then cognitive fatigue will increase as the length of the task increases, and not depend on task difficulty.

Because the three hypotheses are not mutually exclusive and various interactions are possible, the present experiments are somewhat exploratory. Support for any of the competing hypotheses will help to provide information about the manifestation of cognitive fatigue in MS. The accompanying evidence will be useful in identifying whether cognitive fatigue in MS is domain general or domain specific, whether cognitive fatigue in MS depends on high or low cognitive load, and whether cognitive fatigue increases as time increases.

## Materials and Methods

### Participants

Fifty-six right-handed individuals; 24 healthy controls (HC; 16 female); and 32 (30 female) clinically definite (25) MS patients participated. MS participants were at least 1 month from their most recent exacerbation and reported no current corticosteroid use. Disease duration was available for 30 MS participants and was 11.91 (±7.05) years. Disease subtype was available for 29 MS patients; 24 relapsing-remitting, 1 primary-progressive, 3 secondary progressive, and 1 progressive relapsing. The Ambulatory Index (AI) score was available for 27 MS participants and was 2.44 (±2.53) representing mild to moderate disease progression. All participants had self-reported normal or corrected-to-normal visual acuity and normal color vision. Participants with a history of diagnosed psychological and psychiatric problems (i.e., resulting in patient hospitalization for these disorders) including: epilepsy, learning disability, diagnosis of substance abuse/dependence, brain injury, or loss of consciousness (lasting 30 or more minutes) were excluded. MS and HC groups did not differ in the years of education. The HC group was disproportionately Male and MS group was disproportionately Female, and the MS group was older than the HC group at the time of testing (see Table 1). This study was approved by the Institutional Review Board at the Kessler Foundation, and all participants provided informed consent prior to enrollment.

**Table 1:** **Available demographic information and neuropsychological performance characteristics**.

	HC group			MS group				*t*
	Mean	SD	*N*	Mean	SD	*N*	% Impaired	
Age (years)	37.74	11.09	24	48.23	9.66	32		3.71*
Education (years)	16.13	1.96		15.77	2.33			0.6
Percent female	67%			94%				7.02 (*X*^2^)*
DST scaled score	11.91	2.97	23	10.87	4.30	31		1
SDMT z	0.32	1.19	23	−0.71	1.32	31		2.95*
PASAT 2 z	0.01	0.90	23	−0.57	1.13	30		2.05*
PASAT 3 z	0.08	0.81	23	−0.58	1.16	30		2.32*
CVLT-II LDFR z	0.02	1.14	23	−0.40	1.26	31		1.28
BVMT-R DR T	58.43	9.10	23	48.20	13.21	30		3.18*
JLO corrected score	28.05	3.44	22	25.87	5.53	31		1.64
CMDI Total *t*-score	45.47	7.88	18	54.15	8.48	27		3.47*
FSS raw	2.12	0.93	18	5.09	1.47	27	74%	7.63*
MFIS total	9.56	9.06	18	44.86	16.46	28	89%	8.31*
Physical	3.61	3.62	18	21.32	8.44	28	89%	8.4*
Cognitive	5.06	5.09	18	21.00	7.68	28	75%	7.77*
Psychosocial	0.89	1.08	17	4.32	2.06	28	57%	6.52*

*p < 0.05.

*Independent samples *t*-tests comparisons between MS and HC groups. Percent (%) high fatigue calculated based on a cut-off value with scores 1.5 SD greater than the HC mean interpreted as high fatigue. DST, digit span total; SDMT, symbol-digit modality test; PASAT 2, paced auditory serial addition test 2 s; PASAT 3, paced auditory serial addition test 3 s; CVLT-II LDFR, California verbal learning test – II long delay free recall; BVMT-R DR, brief visuospatial memory test – revised delayed recall; T, JLO, judgment of line orientation; CMDI, Chicago multi-scale depression inventory; FSS, fatigue severity scale; MFIS, modified fatigue impact scale total score, and physical, cognitive, and psychosocial subscales*.

### Neuropsychological testing

The following specific neuropsychological tests (and differences between them) were particularly relevant to the present investigation and part of a larger neuropsychological testing session (see Table 1 for additional neuropsychological assessment scores). The MS and HC groups did not differ on WM (Digit Span Total), however, the MS group was significantly impaired on PS [Symbol-Digit Modalities Test (SDMT)]. The MS group reported higher depression (Chicago Multi-scale Depression Inventory) and higher fatigue on the Fatigue Severity Scale and all subscales of the Modified Fatigue Impact Scale. Additionally, because fatigue was the main focus of this study, we computed the percentage of the MS sample that report high fatigue (≥1.5 SDs above the HC mean) on the FSS and MFIS subscales (Table 1).

### Experimental design

The experiment was conducted over two separate testing sessions, within a 2-week time period. Each session involved different cognitive domains; either a PS or WM task. Experiments were conducted concomitantly with an *f* MRI scan (imaging results to be reported separately). The order of testing sessions and order of tasks within the testing sessions were counterbalanced across participants. All participants received all manipulations within subjects. Stimuli were presented using E-prime software and response time (RT) and accuracy was recorded.

### Processing speed

**Figure 1:**
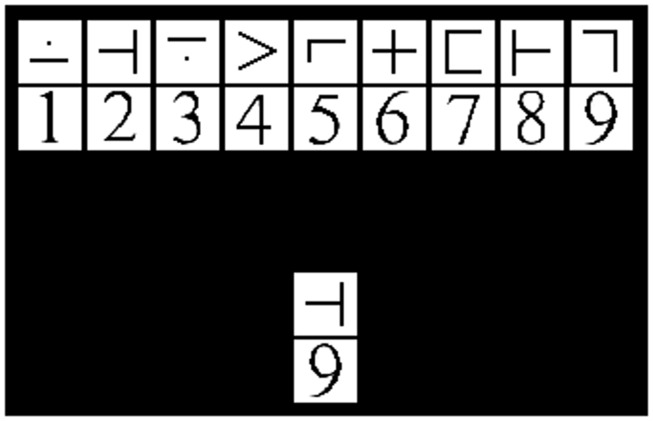
**Example of the stimuli used during the mSDMT**. Target represents a no-match trial.

The modified SDMT (mSDMT) (26, 27) and a visual matching control task were manipulated within participants resulting in high and low cognitive load, respectively. The sessions were separated by a 10-min break in order to allow the participants time to rest and reorient themselves to the new task before beginning the second part of the experiment. The entire experiment consisted of 8 blocks, 4 blocks for each task, and each block consisted of 55 trials. During the mSDMT, participants viewed a 2 × 9 grid of exemplar stimuli (i.e., the key). The upper and lower rows of the exemplar grid contained symbols and digits, respectively. A 1 × 2 grid probe was positioned below the key and participants were instructed to respond “match” or “no-match” as fast and accurately as possible. The match/no-match decision depended on whether the probe corresponded to the exemplar stimuli in the key positioned above (Figure 1). The paired stimuli and probe remained on the screen for 3500 ms. To minimize learning and practice effects, the exemplar symbol-digit combination in the key randomly changed with each trial. In the visual matching control task, participants were presented with the same 2 × 9 grid; however, they responded when the test-probe was a “7.”

### Working memory

The 2-back and 0-back version of the n-back task were manipulated within participants resulting in high and low cognitive load, respectively. The sessions were separated by a 10-min break to allow participants time to rest and reorient themselves before beginning the second half of the experiment. The entire experiment consisted of 8 blocks, 4 blocks for each task, and each block consisted of 65 trials. A series of single letters were sequentially presented and participants responded when the target letter was a “K” (0-back) or when the target letter matched the letter from two trials prior (2-back). Stimuli remained on the screen for 1500 ms.

### Measuring subjective cognitive fatigue

State fatigue (28) was measured one time before the experiment began (establishing baseline) and once after each block (run) using the Visual Analog Scale (VAS) for Fatigue. The VAS is a valid and reliable instrument used to measure self-reported fatigue in MS (29, 30). Participants orally reported how mentally fatigued they felt “right now at this moment,” on a scale of 0–100. This measurement provides an online assessment of fatigue (state fatigue), rather than an estimate of fatigue over an extended period of time (c.f., FSS, MFIS, trait fatigue) (28), allowing quantifications of the level of fatigue resulting from the different tasks across blocks. Additionally, we asked participants to focus on their feelings of fatigue at that moment and disregard prior feelings of fatigue.

### Statistics

Group differences on demographics and neuropsychological performance were evaluated using independent sample *t*-tests. Three separate mixed model ANOVA’s were conducted on the VAS, Accuracy, and Response Time data, respectively, to investigate the effect of the independent variables on the dependent measures. Age, gender, and depression scores were initially included as covariates in all models, however, scores on these variables did not significantly covary, thus the reported analyses described below do not include age, gender, or depression scores as covariates in the models. Pearson correlation coefficients were used to investigate any relationships between subjective (VAS scores) and objective (performance) cognitive fatigue. Alpha was set at 0.05 for all comparisons except where noted. All statistical analyses were computed with IBM SPSS Statistics Release 21.0.0.1.

## Results

### Subjective cognitive fatigue: VAS

**Figure 2:**
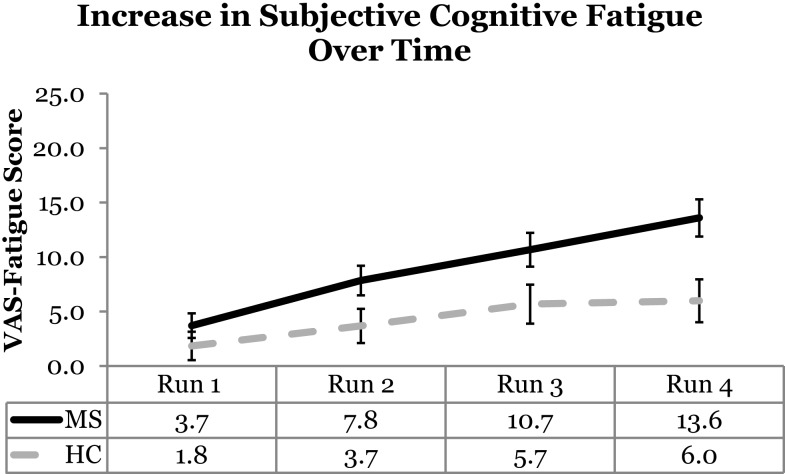
**VAS scores as a function of run and group**. The MS group showed higher fatigue than the HC group across runs. Error bars represent standard error.

Visual analog scale cognitive fatigue measurements taken after each block were subtracted from the initial VAS baseline measurements to control for baseline cognitive fatigue. VAS scores were analyzed using a 2 (Group: MS vs. HC) × 2 (Cognitive Domain: PS vs. WM) × 2 (Cognitive Load: High vs. Low) × 4 (Run: 1, 2, 3, 4) Mixed ANOVA. The main effect of Cognitive Domain was significant, *F*(1,54) = 5.50, *p* = 0.02, η_p_^2^ = 0. 09, with higher VAS scores reported for the PS (*M* = 8.13) than WM (*M* = 5.12) Domain. The main effect of Run was significant, *F*(3,162) = 17.98, *p* < 0.001, η_p_^2^ = 0. 25, with a significant linear trend, *F*(1,54) = 37.18, *p* < 0.001, η_p_^2^ = 0. 41, the VAS scores increased as a function of Run suggesting that subjective cognitive fatigue increased over time. The main effect of Cognitive Load was not significant, *F*(1,54) = 2.53, *p* = 0.12. The main effect of Group was significant, *F*(1,54) = 6.45, *p* = 0.01, η_p_^2^ = 0. 11, with higher VAS scores, and higher reported subjective cognitive fatigue, for the MS group (*M* = 8.95) than HCs (*M* = 4.30). The Run by Group interaction was also significant, *F*(3,162) = 2.71, *p* = 0.047, η_p_^2^ = 0. 05. The significant Run by Group interaction suggests that the MS group showed higher VAS scores (higher fatigue) across runs (Figure 2). This finding supports the Temporal Fatigue Hypothesis.

#### Subjective cognitive fatigue: VAS

##### High vs. low trait fatigue in MS

The MS patients were divided by fatigue group and identified as being either high or low in trait fatigue as measured by the FSS and MFIS. MS patients were classified as high fatigue if their FSS or MFIS score were ≥1.5 SD above the HC mean (see Table 1 and Neuropsychological Testing). The analysis reported above was recomputed using *MS Fatigue Group* as the between subjects variable. This helped to identify whether patients who reported high compared to low trait fatigue differed in their pattern of state fatigue (VAS scores). High trait fatigue patients were compared to low trait fatigue patients using a 2 *MS Fatigue Group* (MS High Fatigue vs. MS Low Fatigue) × 2 Cognitive Domain × 2 Cognitive Load × 4 Run Mixed ANOVA.

The main effect of MS Fatigue Group was not significant and there were no interactions between MS Fatigue Group and any of the other independent variables. This was true when classifying patients’ trait fatigue using the FSS (all *p*’s > 0.40) and when classifying patients’ trait fatigue using the MFIS (all *p*’*s* > 0.13). These supplementary analyses on the quasi variables of high and low fatigue should be interpreted cautiously because the proportion of patients classified as low fatigue was small. The majority of the sample reported high levels of fatigue when classified by the FSS and MFIS trait fatigue measures (Table 1).

#### Subjective cognitive fatigue: VAS

##### Correlation between state and trait fatigue

We also investigated the correlations between measures of trait (MFIS and FSS scores) and state fatigue (VAS scores) in the MS group. The FSS and MFIS scores were positively correlated with each other, *r*(27) = 0.46, *p* = 0.02. Because of the high number of correlational comparisons, alpha was adjusted using a Bonferroni correction for each set of comparisons (alpha = 0.006). None of the correlations between the trait (FSS or MFIS) and state (VAS scores) fatigue measures was significant. Additional correlations were computed between the MFIS *cognitive fatigue subscale* and VAS scores. This was done to determine whether there was a noticeable relationship between state and trait cognitive fatigue using the more specific subscale of the MFIS. No correlations between the MFIS cognitive fatigue subscale and VAS scores reached significance.

The lack of a correlation between the trait and state fatigue measures suggest that trait and state fatigue may be independent or that state fatigue is not well captured by the trait fatigue measures. No observable correlations may also be because the state fatigue measure (VAS scores) captured online cognitive fatigue “right now” and the trait fatigue measures captured general fatigue over the past week (FSS) or past 4 weeks (MFIS). Further, the FSS and MFIS include items that may not directly capture cognitive fatigue, rendering the scores more representative of general fatigue. Even the specific items on the MFIS cognitive subscale seem ambiguous in this regard.

### Objective cognitive fatigue: Accuracy

Due to a programing malfunction, only a subset of behavioral data recorded and was available for data analysis (HC = 12; MS = 18). Accuracy was analyzed using the same 2 × 2 × 2 × 4 Mixed ANOVA. The main effect of Cognitive load was significant, *F*(1,28) = 34.42, *p* < 0.001, η_p_^2^ = 0. 55, with lower accuracy in the high (*M* = 0.93) compared to low (*M* = 0.99) load condition. The main effect of Run was significant, *F*(3,84) = 3.36, *p* = 0.02, η_p_^2^ = 0. 11, with a significant quadratic trend, *F*(1,28) = 4.72, *p* = 0.04, η_p_^2^ = 0. 14. The quadratic trend was driven by low accuracy on the first run and an increase and plateau in accuracy for runs 2, 3, and 4. The main effect of Group was not significant, *F*(1, 28) = 0.19, *p* = 0.67. The Domain × Cognitive Load interaction was significant, *F*(1,28) = 4.19, *p* = 0.05, η_p_^2^ = 0. 13, with accuracy lower for WM under a high load than the other conditions.

#### Objective cognitive fatigue: accuracy

##### High vs. low trait fatigue in MS

Multiple sclerosis Fatigue Group comparisons were not conducted for MS patients because of the missing accuracy data.

### Objective cognitive fatigue: RT

Response time was analyzed for accurate trials only using the same 2 × 2 × 2 × 4 Mixed ANOVA on a subset of the data (HC = 10; MS = 17). The main effect of Domain was significant, *F*(1,25) = 168.36, *p* < 0.001, η_p_^2^ = 0. 87, with slower RTs for PS (*M* = 1237) than WM (*M* = 700). The main effect of Cognitive Load was significant, *F*(1,25) = 188.89, *p* < 0.001, η_p_^2^ = 0. 88 with slower RTs in the High (*M* = 1194) than Low Load (*M* = 742) condition. The main effect of Group was significant, *F*(1,25) = 15.63, *p* < 0.001, η_p_^2^ = 0. 39, with slower RTs for MS (*M* = 1078) than HCs (*M* = 859). The Domain × Group interaction was significant, *F*(1,25) = 4.50, *p* = 0.04, with the MS group showing a larger difference between Domains than HCs. The Domain × Load interaction was significant, *F*(1,25) = 162.86, *p* < 0.001, η_p_^2^ = 0. 87, with the High Load condition of the PS task resulting in substantially slower RTs than the other conditions. This effect was further augmented by the Domain × Load × Group interaction, *F*(1,25) = 6.87, *p* = 0.02, η_p_^2^ = 0. 22, with a larger difference in RTs between the MS and HC groups in the High Load condition of the PS task. The Load × Run × Group interaction was significant, *F*(1,25) = 4.65, *p* = 0.005, η_p_^2^ = 0. 16, along with the four-way interaction *F*(3,75) = 3.28, *p* = 0.03, η_p_^2^ = 0. 12. The MS group was slower during early runs but showed improvement across runs and this was true only in the high Cognitive Load condition.

#### Objective cognitive fatigue: RT

##### High vs. low trait fatigue in MS

Multiple sclerosis Fatigue Group comparisons were not conducted for MS patients because of the missing RT data.

### Correlations between subjective and objective cognitive fatigue

Correlations were computed between the VAS scores and RTs to better understand the relationship between subjective and objective fatigue. Correlations were not computed for accuracy because the tasks did not differ between MS and HCs. After a Bonferroni correction, none of the correlations between VAS scores and RTs reached significance. We further explored these same correlations using only responses from the MS group and found no correlations reached significance.

## Discussion

Irrespective of Cognitive Load, subjective cognitive fatigue increased as the length of the task increased. The present data support the Temporal Fatigue hypothesis over the Cognitive Load hypotheses. There was some support for higher reported fatigue in the PS domain than in the WM domain, however, this was observed in both the MS and HC groups.

Correlations used to investigate the relationship between subjective and objective cognitive fatigue were not significant, suggesting subjective and objective cognitive fatigue are independent and supportive of prior work (9, 14–19). Subjective and objective cognitive fatigue may continuously fail to correlate because behavior and performance may not be the ideal measure of fatigue (9). The often replicated lack of a relationship between subjective and objective cognitive fatigue (9, 14–18) may suggest researchers pursue alternative objective measures of cognitive fatigue. Importantly, cognitive fatigue does not have to result in changes in behavior or performance deficits, thus a relationship may not be supported in large part because the intuitive assumption that cognitive fatigue and performance will be related is inaccurate. Additionally, measurement of trait (FSS and MFIS) and state fatigue (VAS) was uncorrelated suggesting trait measures may not capture state fatigue.

Neuroimaging may be one potential direction that may help identify the mechanisms associated with fatigue. Several investigations highlight the involvement of the fronto-striatal network in cognitive fatigue in a variety of clinical populations, including MS (31). In MS, fMRI studies have also found the fronto-striatal network to be associated with fatigue during task performance (26) and tracked brain activity as a function of on-task fatigue (28). Interestingly, the pattern of activation also appeared to be independent of behavioral performance in that study (28).

The present findings suggest that MS participants experience subjective cognitive fatigue as the time of the task increased, regardless of the cognitive domain, and regardless of the cognitive load associated with the task. To our knowledge, no other studies have made direct comparisons between cognitive fatigue resulting from a PS task compared with a WM task in MS. In past work, Bailey et al. (16) reported higher fatigue during the 1-back component for participants with advanced MS. Cognitive load was arguably higher (2-back) in the present study and there was no difference found in cognitive fatigue between the MS and HC groups. This may be because the Bailey et al. study limited their sample to what they referred to as advanced MS. That is, patients who scored between 7 and 8 on the EDSS. EDSS scores were not available in the present study; however, AI scores were available for most of the MS group. AI scores are highly correlated with EDSS [*r* = 0.89; DeLuca et al. (32)] and the AI scores of the present sample suggested mild to moderate disease severity. The present sample was likely less extreme than the Bailey et al. sample and mainly comprised of relapsing-remitting MS participants. It is possible that the experience of cognitive fatigue is greater at higher cognitive loads during advanced stages of the disease or different disease subtypes. This is one avenue for future work.

Multiple sclerosis participants who experience cognitive impairment [either WM (24) or PS (22, 23) impairments] might be more susceptible to cognitive fatigue during cognitive task performance that is related to the impaired cognitive domain. We could not investigate this hypothesis with the current data. None of the MS participants in the present sample showed impairments in WM and only six MS participants scored 1.5 standard deviations or more below the mean on the SDMT [supporting (22, 23)]. Similar to the current design that manipulated cognitive load, cognitive domain, and time, future work should also differentiate the groups based on cognitive impairment. MS participants with cognitive impairment (WM or PS) should be compared to MS participants without cognitive impairment. The cognitive fatigue profile may differ for these participants.

There are limitations associated with the present study restricting the generalizability of the findings. First, the MS sample and HC sample were disproportionately female and male, respectively and the HC group was slightly younger in age and reported lower depression. After controlling for age, gender, and depression in our main analysis, we found no covariance. Nonetheless, these differences should be kept in mind when making comparisons across studies and generalizing the MS community in general. Second, the duration of the decision screen of the PS task was somewhat longer than the decision screen of the WM task because the PS decision required more time, rendering the PS task somewhat longer than the WM task. Typically MS participants are more familiar with neuropsychological tests, given they may undergo assessment at different intervals as the disease progresses. It is possible that MS patient familiarity with the test procedures increased their overall performance, masking noticeable differences between the HC and MS group. Importantly, familiarity with the different tests remains unknown in this study.

Additionally, the MS group may have had to work harder than the HC group to achieve equivalent performance, and this extra effort resulted in higher fatigue. It remains possible that cognitive fatigue increased as a result of cognitive load or cognitive domain; however, this may have resulted in participants exerting more effort to maintain efficient performance. Such a relationship may show no change in objective performance scores but will show an increase in reported fatigue. The subjective–objective relationship may resemble a complex feedback loop between cognitive effort and cognitive fatigue that goes unnoticed by objective performance-based measures. The relationship may be one whereby cognitive effort results in increases in cognitive fatigue and those increases in cognitive fatigue result in additional cognitive effort – *ad infinitum* – until the cognitive task is discontinued. The present findings cannot directly rule out this complimentary theoretical explanation describing the relationship between subjective and objective cognitive fatigue. It may be possible to disentangle this account in future research if valid and reliable measures of cognitive effort are correlated with cognitive fatigue [perhaps physiological measures of pupillometry will be one viable approach, c.f., Hess and Polt (33)]. Presently, the assumptions associated with this theoretical perspective remain open to further empirical investigation.

## Conclusion

Irrespective of cognitive load, subjective cognitive fatigue increased as a time increased and this was magnified for the MS group. The independence of subjective and objective cognitive fatigue replicates past work in MS. These data suggest a temporal nature of cognitive fatigue in MS. Researchers should consider sustained task length as an important variable to control for when designing and conducting studies investigating cognitive fatigue and consider measuring subjective fatigue at multiple specific intervals. It remains possible that subjective cognitive fatigue may manifest differently in other neurological populations and other MS disease subtypes. This hypothesis will need to be further evaluated in future research.

## Conflict of Interest Statement

The authors declare that the research was conducted in the absence of any commercial or financial relationships that could be construed as a potential conflict of interest.
